# Metabolite, protein, and tissue dysfunction associated with COVID-19 disease severity

**DOI:** 10.1038/s41598-022-16396-9

**Published:** 2022-07-16

**Authors:** Ali Rahnavard, Brendan Mann, Abhigya Giri, Ranojoy Chatterjee, Keith A. Crandall

**Affiliations:** 1 Computational Biology Institute, Department of Biostatistics and Bioinformatics, Milken Institute School of Public Health, The George Washington University, Washington, DC 20052 USA; 2 Department of Microbiology, Immunology, and Tropical Medicine, School of Medicine and Health Sciences, The George Washington University, Washington, DC 20052 USA

**Keywords:** Computational models, Data mining, Viral infection, Bioinformatics

## Abstract

Proteins are direct products of the genome and metabolites are functional products of interactions between the host and other factors such as environment, disease state, clinical information, etc. Omics data, including proteins and metabolites, are useful in characterizing biological processes underlying COVID-19 along with patient data and clinical information, yet few methods are available to effectively analyze such diverse and unstructured data. Using an integrated approach that combines proteomics and metabolomics data, we investigated the changes in metabolites and proteins in relation to patient characteristics (e.g., age, gender, and health outcome) and clinical information (e.g., metabolic panel and complete blood count test results). We found significant enrichment of biological indicators of lung, liver, and gastrointestinal dysfunction associated with disease severity using publicly available metabolite and protein profiles. Our analyses specifically identified enriched proteins that play a critical role in responses to injury or infection within these anatomical sites, but may contribute to excessive systemic inflammation within the context of COVID-19. Furthermore, we have used this information in conjunction with machine learning algorithms to predict the health status of patients presenting symptoms of COVID-19. This work provides a roadmap for understanding the biochemical pathways and molecular mechanisms that drive disease severity, progression, and treatment of COVID-19.

## Introduction

The COVID-19 pandemic continues to make an impact globally as communities reshape their activities due to the spread of various emerging SARS-CoV-2 strains. Despite the generation of multiple efficacious vaccines, our understanding of the factors that contribute to disease severity remains limited^1,2^. A number of observational studies have established a relationship between severe COVID-19 and pre-existing conditions such as Type 2 Diabetes and obesity^3^. Utilizing a multi-omic approach to investigate how comorbidities may contribute to both sides of the virus-host interaction will allow for a molecular-level understanding of the infection and may lead to improved preventive or therapeutic interventions. Omics data sets are highly complex, often containing a high degree of dimensionality and zero-inflation which can complicate analyses that rely on conventional statistical testing. Therefore, as new computational tools are developed to handle omics data, reanalysis of publicly available COVID-19 data may reveal novel findings. Our group, alongside our collaborators, has recently generated several innovative methods for identifying key clusters^4^, molecules^5^, and biological processes^6^ from omics data.

We previously have shown that SARS-CoV-2 genomic variation is independent of host characteristics (e.g., age and gender)^7^. SARS-CoV-2 genome variation over time has introduced new strains with various functional characteristics such as a set of mutations in spike protein, the primary vaccine antigen^8^, and folding conformations in the virus variants and related functions^9^. Host response to infection can be measured by profiling small molecules (metabolites) and large molecules (proteins). For example, 3′-Deoxy-3′,4′-didehydro-cytidine (ddhC), a human antiviral metabolite, was significantly increased in COVID-19 patients^10^, and Gamma aminobutyric acid (GABA) metabolite was suggested as a potential signaling molecule by activating B cells and plasma cells^11^. We aimed to investigate metabolite and protein profiles to develop a comprehensive snapshot of host response and identify potential molecular biomarkers associated with COVID-19 disease severity.

We investigated the factors that influence COVID-19 disease severity by reanalyzing a previously published integrated study of metabolite and protein profiles, epidemiological data, and clinical data^12^. The measurements included a complete blood cell count panel, a comprehensive metabolic test panel, and quantification of 941 metabolites and 894 proteins from serum samples. Metabolite profiling and protein profiling were performed using ultra-performance liquid chromatography-tandem mass spectrometry (UPLC-MS/MS) and stable isotope-labeled proteomics strategy TMTpro (16plex)^13^.

## Results

**Figure 1:**
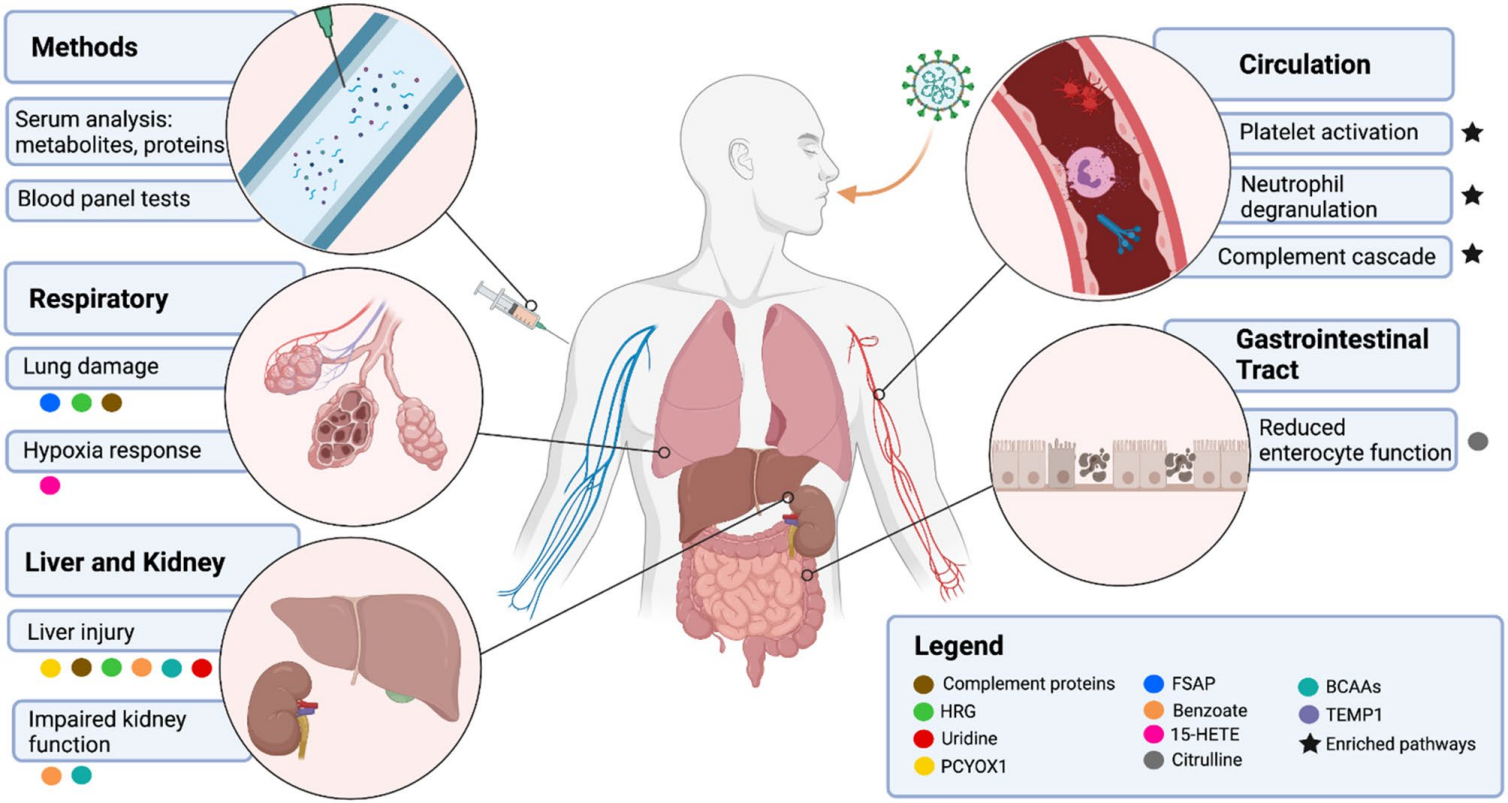
Systemic drivers of COVID-19 associated inflammation. COVID-19 begins as a respiratory tract infection that targets the lung epithelium, but in many severe cases, as the disease progresses, clinical manifestations can span the entire body as a result of systemic inflammation. This includes multisystem abnormalities in biological processes and metabolic functions that may exacerbate the inflammatory response observed in severe cases. An integrated analysis of proteomics and metabolomics data collected from a cohort presenting a range of COVID-19-related health outcomes led to the identification of potential biomarkers within the lungs, liver, gastrointestinal tract, kidneys, and peripheral blood. This analysis provides a deeper resolution of the possible molecular determinants of COVID-19-associated inflammation that are worthy of further investigation. This figure was created with BioRender.com.

Although the mode of transmission for SARS-CoV-2 infection is primarily through the respiratory tract, the effects of COVID-19 can often be observed throughout the body^14^. This is especially the case for severe COVID-19, which results in prolonged systemic inflammation that can damage multiple organ systems^15,16^. The mechanism behind these events can be investigated through the interrogation of soluble factors present within the circulation during infection. This includes proteins which are the downstream products of gene expression and metabolites which are products of biological reactions. Quantification and joint analysis of these molecules may identify the molecular determinants of the aberrant inflammation observed in COVID-19 cases leading to improved diagnostic and therapeutic methods (Fig. 1).

**Figure 2:**
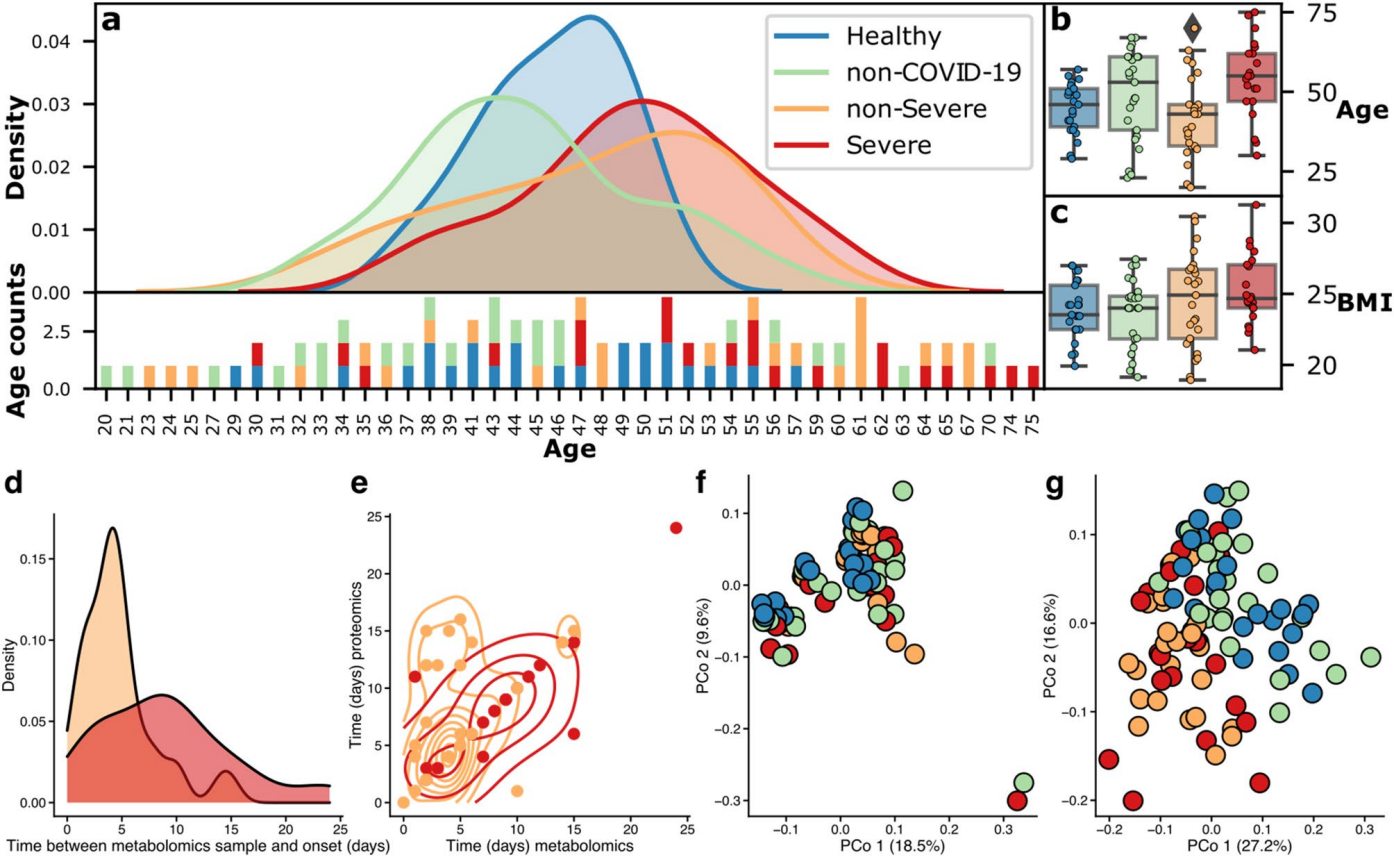
Distribution of study participants by age and health outcome. (**a**) colors in all subplots reflect health status of groups as provided in the legend of subplot a. The sample population is not uniform for age ranges, with a higher proportion of older participants falling into the severe group. (**b**) age has a strong association with health outcomes (*p-value* = 0.001). (**c**) BMI is not associated with health outcomes (*p-value* = 0.1488). For assessing the association of age and gender with the health outcome status, we performed the Kruskal–Wallis test. (**d**) time between disease onset and sample collection for metabolites varies among health status. (**e**) metabolite and protein samples have been collected at different times within individuals and for groups with different health statuses. (**f**) ordination plot using proteins and (**g**) ordination plot using metabolite profiles reveal overall structure among individuals colored by health status. However, the signal is stronger using metabolite profiles measured by omeClust enrichment score^4^ (metabolite enrichment score = 0.26 and protein enrichment score = 0.08) (SFig. 1).

Study design: The original case control study performed by Shen, et al. (Supplementary Table 1) included clinical data (e.g., age, sex, BMI, and symptomes) from 28 Healthy controls, 25 non-COVID-19 participants presenting COVID-19 symptoms but negative for nucleic acid test, 37 non-Severe COVID-19, and 28 individuals with Severe COVID-19. From these groups, metabolite profiling was performed for 96 samples from the following number of individuals: healthy controls (n = 25), non-COVID-19 (n = 25), non-severe COVID-19 (n = 25), and severe COVID-19 (n = 21). Protein profiling was performed for 92 samples from: healthy controls (n = 21), non-COVID-19 (n = 24), non-severe COVID-19 (n = 24), and severe COVID-19 (n = 17). Beginning with an assessment of the clinical data, we found that the health outcome is significantly associated with patient age (*p-value* = 0.001, Kruskal–Wallis test). Pairwise comparisons (Dunn’s test with Benjamini–Hochberg adjustment) revealed that the age distribution was significantly different between the severe and healthy groups (*p-value* = 0.008) (Fig. 2a), and the severe and non-severe groups (*p-value* = 0.002) (Fig. 2b); among infected people, severe COVID-19 is more likely in older individuals. No significant associations were present between health outcome and sex (*p-value* = 0.5308, Kruskal–Wallis test), and health outcome and BMI (*p-value* = 0.148, Kruskal–Wallis test) (Fig. 2c). The time between disease onset and sample collection for metabolites and proteins varies among groups (Fig. 2d) in the study and should be considered in subsequent analyses, especially as the sample collection for proteomics and metabolomics are not at the same time for individuals ((Fig. 2e). Overall protein (Fig. 2f) and metabolite (Fig. 2g) profiles can be explained by clinical information (using omeClust enrichment score^4^) and also by various health outcomes. However, sex seems to have the lowest effect on overall metabolites and protein profiles compared to other clinical variables (SFig. 1).

### Investigation biomarkers of COVID-19 severity and dysfunctions

**Figure 3:**
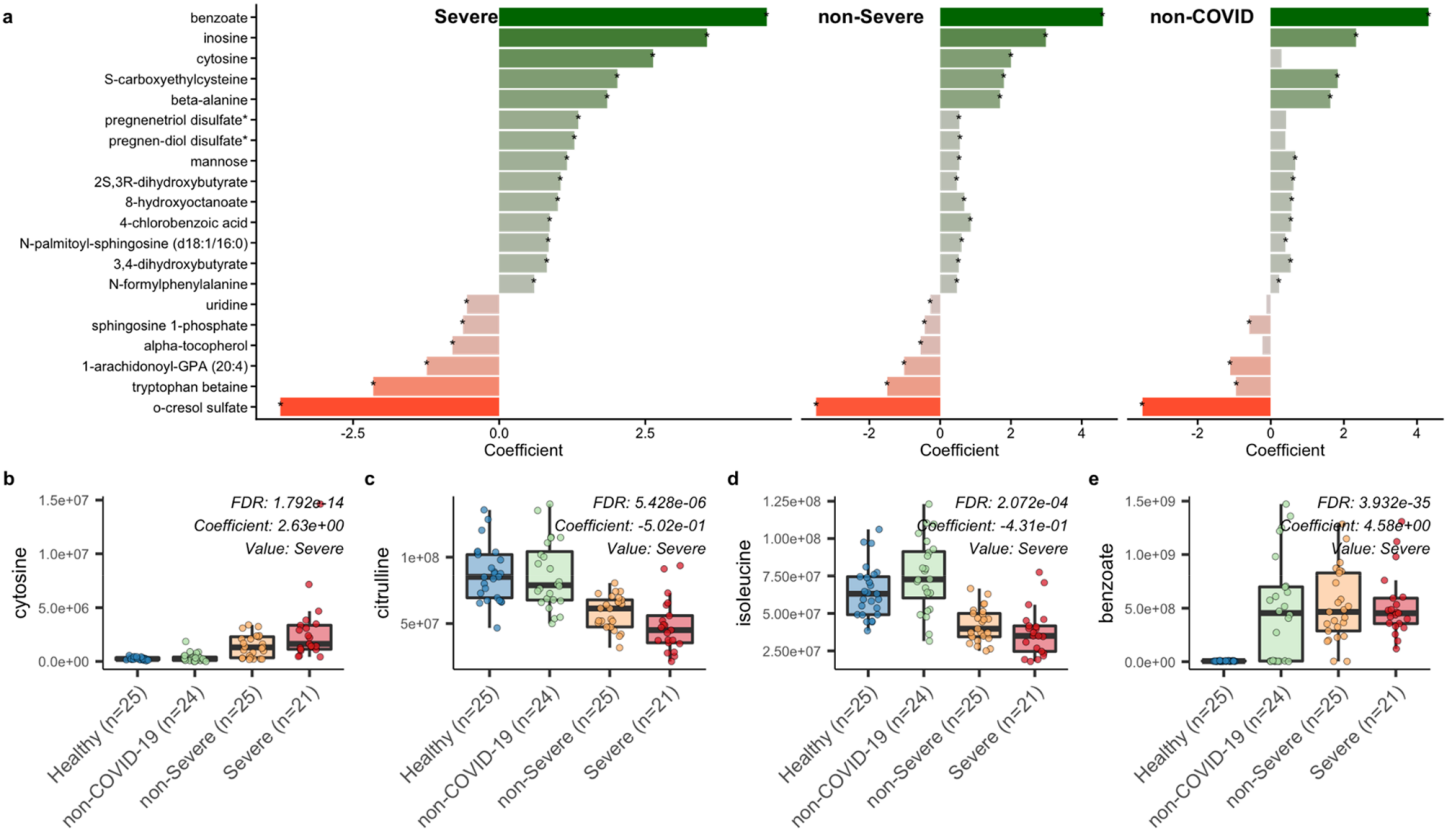
metabolite changes in COVID-19. (**a**) 20 most significant metabolites with lowest q-value (FDR) in comparison of severe group vs. healthy group are shown. Then, the corresponding changes in non-severe and non-COVID-19 for the same metabolites are shown. (**b**,**c**,**d**,**e**) show different patterns we observed among these associations. For example, Cytosine has a higher level in COVID-19 groups vs. non-COVID-19 and has been shown that it can play a biomarker for COVID-19 diagnostics.

**Figure 4:**
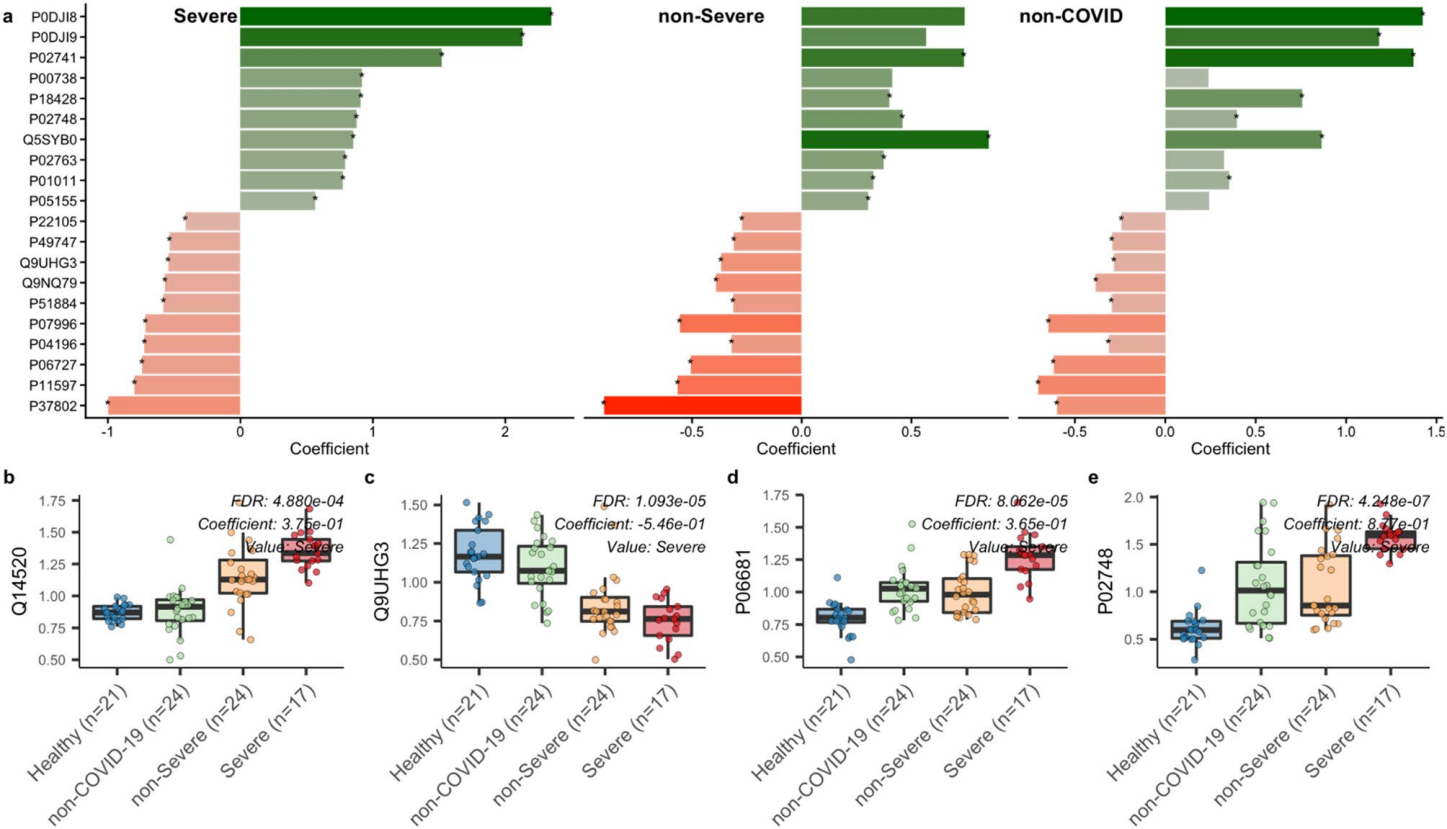
protein changes in COVID-19. (**a**) 20 most significant metabolites with lowest q-value (FDR) in comparison of severe group vs. healthy group are shown. Then, we show the corresponding changes in non-severe and non-COVID-19. (**b**,**c**,**d**,**e**) show different patterns we observed among these associations.

We identified important proteins (Fig. 3a) and metabolites (Fig. 4a) based on significant differences observed among the health outcome groups. We further tested if the associated molecules belong to enriched metabolic pathways. This analysis was conducted using a generalized linear model adjusted for age, sex, and BMI as confounding factors of health outcome. We discuss potential biomarkers identified by our analysis that are biologically relevant during COVID-19 infection. Based on existing literature, these molecules may contribute to the rampant inflammatory response and multiple tissue dysfunction spanning the lung, liver, kidneys, and gastrointestinal tract that have been observed in cases of COVID-19.

### Imbalance of serum nucleic acids

Viruses have adapted over time to hijack cellular machinery and resources for their own replication. Consequently, homeostatic synthesis and recycling of nucleobases may be disrupted in favor of producing new copies of the viral genome. We found that cytosine levels are elevated in the COVID-19 groups as compared to the non-COVID-19 (Fig. 3b) and healthy (coefficient = 2.6, *p-value* = 9.7E-18) groups. This finding is consistent with a similar study analyzing metabolite profiles of COVID-19 patients, which found cytosine to be the distinguishing feature that determined infection status^17^. It is hypothesized that changes in levels of cytosine are critically involved with RNA virus evolution, including SARS-CoV-2^18^. Notably, the underrepresentation of cytosine within the SARS-CoV-2 genome suggests an alternative role for this metabolite beyond the synthesis of viral RNA. While it is unclear why cytosine levels are higher in COVID-19 patients, this finding points towards cytosine as an effective biomarker for COVID-19 infection.

Our results indicate that uridine levels are lower in the COVID-19 groups as compared to the healthy and non-COVID-19 groups. Uridine is a biologically dynamic metabolite that is critical to the synthesis of RNA and glycogen^19^. Circulating uridine levels are typically high in healthy individuals but tend to undergo short term fluctuations in response to diet. Changes in synthesis are tightly regulated by the liver along with the adipose tissue^20,21^. Abnormal liver function appears to be a commonality between SARS-CoV^22,23^ and SARS-CoV-2 infection^24–29^. Therefore, liver impairment may mediate the lower uridine levels observed in COVID-19 patients. The functional consequences of this reduction are also unclear. In an animal model of pulmonary fibrosis, it was shown that uridine supplementation has anti-inflammatory and anti-fibrotic effects^30^. While additional studies are needed, a similar therapeutic strategy may mitigate prolonged inflammation within the lungs that leads to eventual injury and disruption of the epithelium^31^.

### Multi-organ dysfunction

Citrulline, an important amino acid metabolite in the urea cycle^32^, is depleted in the severe (*coefficient* = -0.03974, *p-value* < 0.0001) and the non-severe COVID-19 groups (*coefficient* = -0.0219, *p-value* < 0.0001) compared to the healthy group (Fig. 3c). The depletion of citrulline in COVID-19 patients has been associated with gastrointestinal symptoms and systemic inflammation^33^. Since citrulline is produced by enterocytes within the small intestine, low citrulline levels can be indicative of reduced enterocyte function and mass^34^. Given that enterocytes express ACE2^35^, a host receptor that is recognized by the 2019 coronavirus^36^, it is possible that the presentation of gastrointestinal symptoms^37^ and the lowered citrulline levels in the COVID-19 patients is a result of enterocyte damage via viral infection.

Levels of branched-chain amino acids (BCAAs) leucine, isoleucine, and valine are lower in the COVID-19 groups (Fig. 3d) as compared to the healthy and non-COVID-19 groups (*p-value* = 8E-06 coefficient -0.4 for leucine in severe COVID-19 vs. healthy group). BCAAs play a critical role in protein anabolism^38^, lowered BCAA levels are often observed in various conditions, including liver cirrhosis^39^, urea cycle disorders^40^, chronic renal failure^41,42^, and impaired renal function^43^. Thus, our finding is congruent with the impaired renal function observed in severe COVID-19 cases^44^.

Unlike citrulline and BCAAs, benzoate levels are elevated in the COVID-19 (*coefficient* = 4.5, *p-value* = 8.5E-40) and non-COVID-19 (*coefficient* = 4.3, *p-value* = 4.6E-39) groups compared to the healthy group (Fig. 3e). Benzoate, in the form of sodium salt, is used as a preservative for foods and drinks^45^ and a treatment in urea cycle disorders^46,47^. The metabolism of this compound is directly regulated by the liver and kidneys^48–50^. Benzoate has been found to have both proinflammatory and anti-inflammatory activities. In an in-vitro study with a colon cancer cell line, sodium benzoate was able to induce apoptosis and activate NF-kB^51^, a transcription factor critical for the expression of proinflammatory genes^52^. Conversely, a review of animal models of multiple sclerosis highlighted the anti-inflammatory functions of sodium benzoate, which include promoting the differentiation of anti-inflammatory Th2 cells, increasing the number of regulatory T cells, and reducing the expression of certain proinflammatory molecules such as TNF-alpha and IL-1beta^53^. Therefore, it is not clear if the elevated levels of benzoate in the COVID-19 and non-COVID-19 groups are indicative of a shared biological phenomenon. The high benzoate levels could be reflective of the body’s proinflammatory response to infection. Alternatively, damage to the liver and kidneys due to COVID-19 infection could be disrupting benzoate metabolism, resulting in a backup of benzoate.

Hyaluronan-binding protein 2 or factor VII activating protease (FSAP, protein ID Q14520, Fig. 4b) is a binding protein in the human plasma that is expressed in the liver, kidney, and pancreas^54^. It is known to activate coagulation factor-VII^55^ and urokinase single-chain plasminogen activator^54^. We found higher FSAP levels in the COVID-19 groups compared to the non-COVID-19 groups and associations between FSAP and citrulline, and FSAP and uridine in block-wise association testing (SFig. 2). Several in vitro, as well as patient-based studies, have established a link between FSAP levels, inflammation, and disease. FSAP levels are upregulated in lung endothelial cells that have lipopolysaccharide-induced acute lung injury^56^ and in the inflamed lungs of patients with acute respiratory distress syndrome^57,58^. Increased FSAP levels in plasma are also associated with other pathologies such as symptomatic carotid stenosis^59^, acute coronary disease^60^, and ischemic stroke^61^. In vitro studies have shown that FSAP can activate inflammation pathways in non-immune cell populations such as smooth muscle and endothelial cells^62^ as well as NF-kB mediated proinflammatory cytokine production in myeloid cells^63^. Elevated FSAP levels in the COVID-19 groups could be indicative of systemic inflammation that increases the risk of lung injury and cardiovascular issues.

Prenylcysteine oxidase 1 (PCYOX1 protein, protein ID Q9UHG3, Fig. 4c) is responsible for breakdown of prenylcysteines to cysteines and a C-1 aldehyde^64,65^. It is expressed ubiquitously, but the only expression in the liver leads to its incorporation into lipoproteins^66^; as such, it is associated with very low-density lipoproteins^67^ explaining its presence within the plasma. PCYOX1 levels are depleted in the COVID-19 groups compared to the healthy and non-COVID-19 groups, and the protein is associated with sphingosine-1-phosphate in block-wise association testing. Lower levels of PCYOX1 protein have been observed in mice lacking the interstitial cells of Cajal (ICC) in the gastrointestinal tract^68^ and in mice models of liver injury and dysfunction^66,69–71^. The lower levels of PCYOX1 protein seen in the COVID-19 groups may contribute to the liver dysfunction associated with COVID-19^72^.

### Complement activation

**Figure 5:**
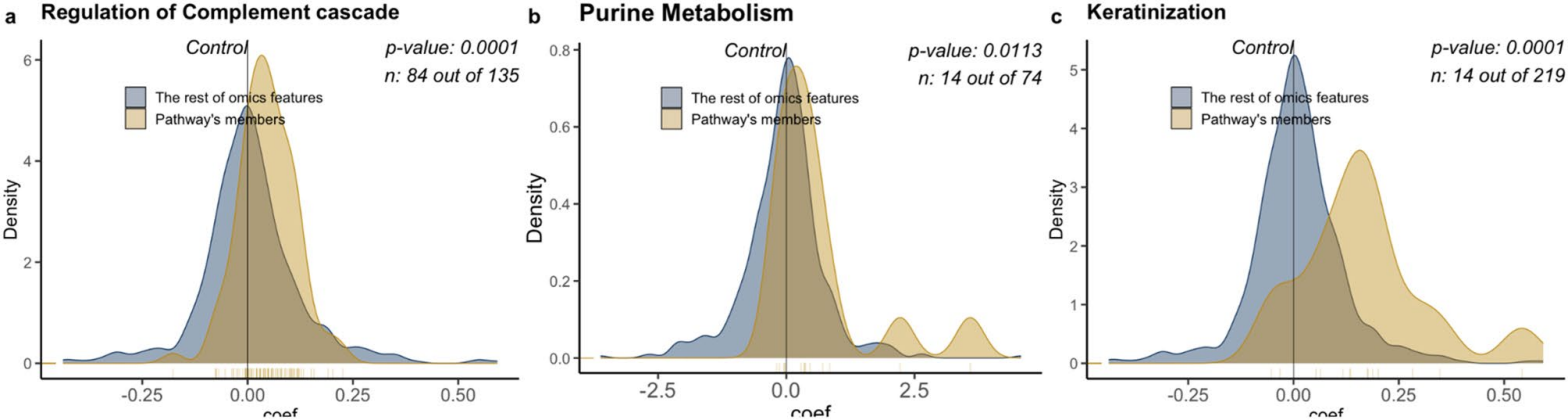
Enrichment pathway between severe group and healthy group. Pathway enrichment analysis was performed for metabolite profiles and protein profiles separately. Each metabolite (protein) assigned a rank based on coefficient from testing severe group vs. healthy group using generalized linear models. We applied our *omePath* tool with a Wilcoxon signed rank test and HMDB database^120^ as the reference for metabolite pathways and the Reactome pathway database^121^ with Physical Entity (PE) class for Uniprot to all levels of the pathway hierarchy mapping file. (**a**) Regulation of Complement cascade pathway using protein data was significantly enriched in the COVID-19 positive patients, with non-severe health outcome compared to the healthy group. (**b**) Purine Metabolism pathway using metabolite data was significantly enriched in COVID-19 positive patients with the severe health outcome compared to the healthy group. (**c**) Keratinization pathway using protein data was significantly enriched in COVID-19 positive patients with the non-sever health outcome compared to the healthy group.

The complement system involves a protein cascade that is typically classified as either the classical lectin or alternative pathways^73–75^. This system plays a major role in B lymphocyte regulation, inflammation, and host protection^76^, and consequently has been associated with proinflammatory actions and diseases^77^. We found levels of the complement component 2 (C2, protein ID P06681, Fig. 4d) and complement component 9 (C9, Protein ID P02748, Fig. 4e) proteins were elevated in the severe and non-severe COVID-19 groups as compared to the healthy group. Briefly, C2 is a protein that forms a short lived complex with C4b to cleave the C3 protein into C3a and C3b^78^, and C9 is involved in the formation of a pore-like membrane attack complex associated with bacterial cell lysis^79^. In addition to the individual components of the complement system, pathway enrichment analysis revealed that the pathway representing complement cascade regulation was significantly enriched between the healthy and other three groups, with the greatest difference found between the severe COVID-19 and healthy groups (Fig. 5a).

Our findings are in agreement with other studies that have suggested the complement system plays a critical role in COVID-19 pathogenesis^74^. For instance, COVID-19 spike proteins have been shown to activate the complement system via the alternative pathway^80^, and evidence of complement system activation correlating with respiratory problems in hospitalized COVID-19 patients^81^. It has also been suggested that unregulated activation of the complement system due to viral presence in the lungs can contribute to organ failure and death^73^. Given that C2 is expressed at higher levels in the liver and lungs, and C9 expression is restricted to the liver^82^, our finding of elevated levels of C2 and C9 proteins in the COVID-19 groups could be indicative of the unregulated activation of the complement system in these organs. Currently, it is believed that the complement system has a contradictory role in COVID-19 infection where it is beneficial in mild or asymptomatic cases and harmful in severe cases^75,83^.

In contrast to the complement component proteins, histidine-rich glycoprotein (HRG, protein ID P04196) levels are heavily depleted in the severe COVID-19 group as compared to the other groups. HRG is a plasma protein that is involved in many biological processes, including immune system regulation, cell adhesion, angiogenesis, and coagulation^84^. HRG inhibits the formation of insoluble immune complexes^85^, which are involved in the host's immune response against foreign substances^86^; when the immune system does not clear these complexes, they can deposit in tissues and activate the complement system inflammation^86^. HRG can also enhance complement activation on necrotic tissues^87^ and is directly involved in clearing of apoptotic^88^ and necrotic cells^89–91^. Low HRG levels have been observed in patients with advanced lung cancer^92^ and advanced liver cirrhosis^93^. Therefore, our findings of depleted HRG levels could be indicative of organ damage in the severe COVID-19 patients, similar to what has been observed in other pathologies.

### Inflammation

15-HETE is a metabolite produced when arachidonic acid is oxygenated by arachidonate 15-lipoxygenase^94^. It is associated with inflammation and can display either pro-inflammatory or anti-inflammatory effects^95^. However, the anti-inflammatory effects of 15-HETE are more well-studied and include inhibition of leukotriene B4 action on polymorphonuclear neutrophils (PMN)^96^ and the migration of PMN in response to cytokines^97^. We found severe depletion of 15-HETE levels in the COVID-19 groups compared to the non-COVID-19 and healthy groups. The depletion of 15-HETE and subsequent loss of anti-inflammatory signals could contribute to the heightened inflammation seen during COVID-19 infection^98^. Interestingly, 15-HETE also plays a role in promoting pulmonary vascular remodeling during hypoxia by exerting pro-angiogenic effects^99,100^. A reduction in serum 15-HETE levels would suggest that this metabolite does not directly contribute to the cardiovascular dysfunction observed in severe COVID-19 cases.

To date, severe COVID-19 has been associated with an increase in the immediate and long-term risk of thrombosis and coagulation abnormalities^101,102^. This has primarily been attributed to prolonged overactivation of platelets and high levels of neutrophil degranulation, both of which represent significantly enriched pathways in our analysis of this group. Increases in platelet mediated prothrombosis are typical in response to many invading pathogens. However, SARS-CoV-2 infection in particular triggers significant changes in platelet gene expression and aggregation. Furthermore, excessive platelet activation from COVID-19 leads to alterations in innate immune responses that contribute to thrombotic events. This includes the accumulation of platelet-monocyte complexes, which directly express high tissue factor levels, which directly increases the risk of clotting^103^. High plasma levels of platelet factors also contribute to dysregulated neutrophil responses, such as the excessive formation of neutrophil extracellular traps and degranulation^104,105^. In addition to their direct detrimental role to cardiovascular health, these factors may also exacerbate systemic inflammation leading to damage within the tissues.

Pathway level analysis may also be beneficial for identifying overarching mechanisms that have a role in regulating rampant inflammation. Collectively, the purine metabolism pathway is significantly enriched within the COVID-19 groups compared to the healthy control (Fig. 5b). Cell-free purine derivatives such as ATP and adenosine are associated with cellular stress and exert potent immunomodulatory effects. Release of ATP into the extracellular space and subsequent binding to purinergic receptors P2X and P2Y lead to the induction of inflammation including the activation and chemotaxis of phagocytes and memory T cells^106^. Sustained levels of extracellular ATP trigger the expression of ectoenzymes on the surface of immune cells that convert ATP to adenosine. Binding of adenosine to cognate purinergic receptors tempers inflammation by reducing neutrophil chemotaxis and platelet aggregation in addition to promoting wound healing via the release of vascular endothelial growth factor (VEGF) by macrophages and dendritic cells^107^. Under hypoxic conditions, hypoxia inducible factors HIF-α and HIF-β can alter adenosine metabolism in order to protect tissue from further damage brought about by prolonged inflammation^108^. Acute lung injury that occurs during severe cases of COVID-19 may trigger such pathways leading to the observed enrichment in purine metabolism.

### Fibrosis/keratinization

Lumican (protein ID P51884) regulates fibril assembly and stromal collagen matrix assembly^109^. In mice models, it has been found that lumican is critical for host immune innate response^110,111^, and its deficiency has been associated with cardiomyocyte hypertrophy^112^. In a study of Nepalese children, lumican levels were negatively associated with levels of α-1-acid glycoprotein^113^, an acute phase protein that increases during inflammation, infection, or injury to tissues^114^. We found that lumican levels were depleted in the COVID-19 groups compared to the non-COVID-19 and healthy groups and is also associated with some glycerophospholipids in block-wise association testing. Thus, lower lumican levels may act as an additional biomarker of rampant inflammation in the COVID-19 groups. Alternatively, lower lumican may lead to disruption of the collagen and fibril assembly pathways as a consequence of infection.

Analyses of lung tissue from mechanically ventilated or recently deceased patients with severe COVID-19 revealed high levels of inflammatory infiltrate and fibrotic markers indicative of extensive epithelial and alveolar damage^115,116^. Identification of additional biomarkers could facilitate diagnosing the severity of lung injury prior to the induction of respiratory failure. Our analysis identified significant alterations in processes that maintain cell or tissue structure, including enrichment of proteins involved in the keratinization pathway (Fig. 5c). Keratins play a vital role in both maintaining the structural integrity of the epithelium and promoting intracellular signaling to mediate wound healing. Cytoskeletal remodeling by keratin intermediate filaments can occur under excessive shear stress or in response to hypoxia^117–119^. The significance of keratinization within the context of COVID-19 has yet to be investigated but may provide insights into the extent of lung damage that occurs in severe cases.

### Correlations between clinical data, omics data, and health outcome

We next examined associations between clinical biomarkers from panel tests and metabolites and proteins from the omics data in the context of COVID-19 severity.

*Glucose:* Severe COVID-19 patients had significantly higher levels of glucose compared to non-COVID-19 patients (*coefficient* = 0.497, *p-value* = –1.2E-05) matching previous studies^122–125^. While the direct impact of COVID-19 infection on glucose levels remains to be elucidated, inflammation may be responsible for the differences in glucose levels observed between the groups. Okin and Medzhitov found that sustained inflammation can lead to elevated glucose levels in the plasma^126^. Alternatively, IFN-gamma production in response to viral infection has been shown to induce insulin resistance^127^ and subsequent higher glucose levels.

In addition to its association with COVID-19 severity, we also found some correlations between glucose and metabolites such as citrulline and uridine. Both of these metabolites were severely depleted in the COVID-19 groups compared to the non-COVID-19 group. While it is unclear if there is a biological connection to these relationships, the metabolism of glucose has been linked with the metabolism of uridine and citrulline. Specifically, uridine has been shown to induce glucose uptake by skeletal muscles^128^ and increased levels of citrulline in the plasma is associated with a reduction in glucose production^129^. Regardless of the biological significance, the correlation of uridine and citrulline with glucose points towards these metabolites being modest candidates for COVID-19 severity biomarkers.

*C-Reactive Protein (CRP):* We found CRP levels to be lower in the non-severe COVID-19 group compared to the non-COVID-19 group (*coefficient* = -−.15 *p-value* = 0.0001), whereas an opposite trend appears when compared to the severe COVID-19 group. This reinforces what has been established in previous studies^124,130–133^. CRP plays a critical role in inflammation and response to infection via the complement pathway and cytokine production^134–136^. Thus, our finding of increased CRP levels in the severe group is in agreement with previous studies that suggest elevated inflammatory markers including procalcitonin, D-dimer, and lactate dehydrogenase^137^ are associated with COVID-19 disease severity.

Similar to glucose, CRP has some metabolic and protein correlates which may be able to serve as novel biomarkers for COVID-19 severity. Specifically, CRP is positively correlated with kynurenine and lipopolysaccharide-binding protein (LBP) (SFig. 3). Kynurenine as a positive correlate of CRP is expected due to its involvement in inflammation and immune activation in various disease contexts^138,139^. Additionally, within COVID-19, kynurenine has been found to be positively correlated with proinflammatory cytokines^140,141^, and activation of the kynurenine pathway has been observed in COVID-19 patients^142^.

The correlation between CRP and LBP is likely a product of these molecules’ role in inflammation. LBP has been shown to be increased in patients with inflammatory conditions like systemic inflammatory response syndrome^143^. Additionally, previous studies have shown that LBP is associated with inflammation markers, including CRP, in patients who have undergone hemodialysis^144^ and in patients with conditions such as acute respiratory distress syndrome and inflammatory bowel disease^145,146^.

*Monocyte:* We found monocyte levels to be significantly decreased in the severe COVID-19 group (*coefficient* = -0.25 *p-value* = 0.04). Based on a meta-analysis of COVID-19 studies involving severe and non-severe patients, lower monocyte counts have been observed as part of a larger trend of immune dysregulation^147^.

*Salicylate:* When investigating associations across all groups, we observed a positive relationship between Monocyte counts and Salicylate (*coefficient* = 1.64 *p-value* = 0.0001). Salicylate is commonly found in non-steroidal anti-inflammatory drugs, including aspirin. Sodium salicylate has been shown to have potent effects on limiting monocyte migration, expression of inflammatory cytokines, and preventing proliferation^148–150^. Whether or not there is a prophylactic benefit to administering aspirin to limit COVID-19 infection is currently the subject of debate^151–153^. A positive association between salicylate and monocyte counts seems to indicate that other factors, either directly related to infection or other forms of treatment, are responsible for the decrease in monocytes observed in patients with severe COVID-19.

*Sphingomyelin:* An additional positive correlation was found between monocyte counts and sphingomyelin (*coefficient* = 0.91 *p-value* = 0.002). Sphingolipids are a class of membrane-associated molecules that play an important role in cell-to-cell interactions and intracellular signaling within the immune system^154^. Sphingomyelin is cleaved by sphingomyelinases to produce ceramide which induces a signaling cascade that can lead to the differentiation, proliferation, apoptosis, or cytokine secretion by select immune cell populations. It was recently shown that neutral sphingomyelinase 2 can cause monocyte migration and secretion of inflammatory cytokines in response to soluble TNF-α ^155^. Elevated TNF-α has been associated with both obesity and severe cases of COVID-19^156^. Most of the COVID-19 biomarkers and their related pathways reported in our study are novel, and some have already been discussed previously^157^ (Supplementary Table 2).

### Group-level correlations between proteins and metabolites

Block-wise association testing was performed to find associations among clusters of metabolites and proteins (Methods). Block 20 in particular shows that 5-methyluridine, citrulline, choline, and uridine are jointly associated with the C4b-binding protein chains (alpha and beta) and Vitamin-K dependent protein S. Inflammation may explain the observed association between the proteins and most of the metabolites in block 20. The C4b-binding protein is involved with the inhibition of the classical and lectin complement system pathways^158,159^. The Vitamin-K dependent protein S complexes with the C4b-binding protein and can modulate the complement regulation activities of C4b-binding protein^160^. Collectively, both of these proteins co-occur due to their involvement in inflammation via complement system regulation^77^. Similarly, there are links between inflammation and three of the four metabolites in the block. Low plasma citrulline levels are known to be associated with systemic inflammation^33^, and uridine is linked with anti-inflammatory effects in an animal model^30^. Furthermore, choline is known to be inversely associated with inflammatory marker levels^161^.

### Deep learning techniques accurately predict disease severity

**Figure 6:**
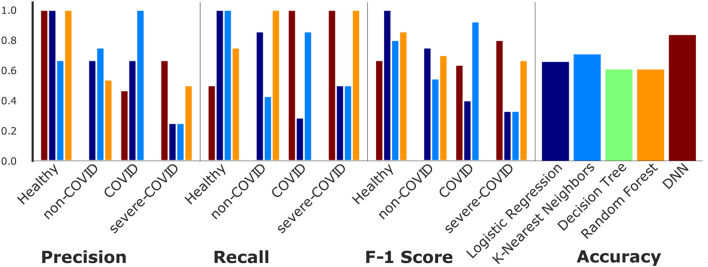
Precision, Recall, and F-1 Score for various ML techniques used for the various groups. The precision of DNN outperforms other methods since it can detect severe-COVID-19 better than the rest of the methods. We also see the same trend in recall since it correctly detects the severe-COVID-19 and COVID-19 with a higher degree of certainty. A model with a low false positive case is better when used for prediction.

We used four machine learning (ML) approaches for disease severity prediction, including deep neural networks (DNN), k-nearest neighbors (KNN), Random Forest (RF), and Logistic Regression (LR) (SFig. 4). We have compared the performance of all the models using precision, recall, F-1, and accuracy metrics (Fig. 6). Accuracy highlights the proportion of true positive in the sum of true positive and false positive. The precision, recall, and f-1 score deals with the false negative, which portrays the versatility and robustness of the model.

DNN outperformed all other methods with an accuracy score of 81.78% when trained using the metabolomics data and clinical data (e.g., age and gender). The other ML processes that were used had accuracy in the range of 60–70%. A higher F-1 score suggests better efficiency of models, which in turn means that the number of false positives is less, thus better prediction.

## Discussion

Integrative analysis of multi-omics enables a more accurate and comprehensive understanding of biological activities at the molecular level. Utilizing omics data can provide finer resolution for identifying the specific molecules or processes that distinguish severe cases of COVID-19. This analysis holds the potential to not only improve our understanding of COVID-19 pathogenesis, but may also lead to improved diagnostic and therapeutic avenues. In this study, we used protein and metabolite profiles from a cohort of donors with varying COVID-19 and health statuses to measure the changes across groups after appropriate adjustment for data properties such as zero inflation and confounding factors. We followed up with pathway enrichment analysis to provide context at a functional level and then investigated associations between significantly different omics features (i.e., protein and metabolite) and clinical information (metabolic panel and complete blood count). Lastly, we utilized our findings to investigate the predictive potential of several machine learning algorithms benchmarked against conventional logistic regression to determine which model is best suited for diagnosing the status of COVID-19 infections.

Phenotype association testing revealed significantly altered proteins and metabolites based on health status (Supplementary Table 3). We found extensive evidence for systemic dysregulation of metabolic processes that may contribute to the varied clinical manifestations observed in severe cases of COVID-19 (Supplementary Table 4). This was reflected in aberrant levels of basic organic molecules that influence viral replication and immune responses, such as cytosine and BCAAs (Fig. 3d). Additionally, although COVID-19 begins as a respiratory tract infection, multiple organs including the gastrointestinal tract, liver, and kidneys can be heavily impacted during infection. Determining if additional organ damage is a product of direct infection, overactive inflammation, or a side effect of treatment remains the subject of investigation. For example, we found lower levels of citrulline in severe cases of COVID-19 (Fig. 3d), indicative of gastrointestinal dysfunction. Whether or not the significant reduction in citrulline is caused by a loss in intestinal enterocytes from direct infection or an alternative mechanism highlights the merit of pursuing these questions. Similarly, over half of the ten proteins and metabolites outlined in our results are directly implicated in severe liver impairment (Fig. 2). Several of these markers were also identified in the original analysis conducted by Shen et al., which revealed alterations in liver-derived acute phase proteins (CRP) and components of the complement cascade. Similarly, both studies found a reduction in key biological processes such as amino acid metabolism (BCAAs) and metabolic intermediates of the urea cycle (citrulline). These similarities underscore the potential damage or dysfunction of the liver during severe COVID-19 cases. While this may potentially be explained by the aggressive use of antiviral and anti-inflammatory drugs that possess hepatic toxicity, the influence of pre-existing medical conditions and behavioral changes associated with the pandemic cannot be discounted^162^.

Despite the development of prophylactic vaccines, the threat of emerging variants necessitates the exploration of additional measures that can limit disease severity. The identification of biomarkers associated with disease severity may be directly translated into repurposing FDA-approved drugs for the treatment of COVID-19. Our analysis revealed a positive correlation between severe COVID-19 and serum levels of glucose (SFig. 3). Therefore, use of glucose-lowering agents such as metformin or glucagon-like peptide-1 receptor agonists may represent an alternative treatment option in addition to the use of antiviral compounds^163,164^. Several observational studies have found positive associations between metformin use and improved mortality rates. Although it has also been demonstrated that metformin can directly inhibit replication of several viruses and therefore additional studies are required to determine the mechanism of action within the context of COVID-19^165,166^.

Pathway enrichment analysis identified pro-inflammatory elements within the circulation as potential etiological agents for multi-organ damage (Fig. 5). Overactivation of platelets and neutrophils contributes not only to thrombotic events but may also give rise to tissue damage in a low oxygen environment. Similarly, persistent activation of the complement system by components of SARS-CoV-2 or other factors may further increase damage to vital organs. By employing novel computational tools that handle complex multi-omics data, we were able to highlight metabolites, proteins, and pathways that distinguish COVID-19 based on infection status and severity. Our analyses identified new potential biomarkers or therapeutic targets worthy of further investigation. Inclusion of additional paired omics data, including metagenomics, single-cell RNA sequencing, transcriptomics, and viral genomics, can provide a better picture of disease pathogenesis and host response to infection and co-infections. Moving forward, a well-designed longitudinal measurement of omics can provide a deeper understanding of both the short and long term effects of infectious diseases, including COVID-19.

## Materials and methods

### Study design and data

All methods were performed in accordance with the relevant guidelines and regulations as described by the authors of the original study^12^. The metabolomic data were from 28 patient cases with severe COVID-19 who were matched to controls based on certain epidemiological features. The matched controls included 28 healthy persons, 25 patients without COVID-19 exhibiting clinically similar signs as COVID-19 patients, and 25 patients with non-severe COVID-19 (Fig. 2a). Proteomic data were available from 17 participants with severe COVID-19, 21 healthy controls, 24 individuals with non-COVID-19, and 24 donors with non-severe COVID-19. Serum samples were obtained a few days after admittance into the hospital. For a small number of cases, serum samples were collected at a later stage of the disease. Twelve clinical measurements were obtained for the COVID-19 and the non-COVID-19 groups but not for the healthy groups; the measurements included a complete blood cell count panel as well as a comprehensive metabolic panel of tests. 941 metabolites and 894 proteins were quantified from the 83 serum samples. Metabolite- and protein- profiling were performed using ultra-performance liquid chromatography-tandem mass spectrometry (UPLC-MS/MS) and stable isotope-labeled proteomics strategy TMTpro (16plex)^13^.

### Data preparation

The study’s aim was to predict health outcome status and discover important features using proteomics and metabolomics data and clinical information. Metabolomic and proteomic data from the original study were downloaded from ProteomeXchange Consortium (https://iprox.org/) by searching for the Project IDs: IPX0002106000 and IPX0002171000. Before feeding the publicly available dataset^12^ into machine learning algorithms, various data cleaning steps were taken. The steps included removing any columns of the 942 metabolites and 640 proteins with missing values across samples and imputing missing BMIs for healthy individuals based on the optimal BMI of Chinese people^167^. We removed other clinical information like platelet counts, etc., since they were only present for the unhealthy patients and not for the healthy subjects. Removing missing values resulted in a dataset that included 404 metabolites and 374 proteins. We also normalized the dataset based on the min–max function available in the *sklearn* package^168^. The cleaned and normalized dataset was split into training (80% of data) and testing (20% of data) subsets to train and test various prediction algorithms, including Logistic Regression, Random Forest, K-Nearest Neighbor, Decision Tree, and Deep Neural Network.

### Omics community detection and prioritizing metadata

We applied *omeClust*^4^ to detect underlying clusters from metabolites profiles and protein profiles independently. *omeClust*, in addition to detecting clusters (communities), also provides an enrichment score for each metadata to measure the potential influence of each metadata on detected structure (clusters). *omeClust* first discretizes metadata; and then calculates enrichment score as normalized mutual information between cluster labels and discretized metadata.

### Multivariate association testing

We used multivariate association testing with considering noisy, sparse (zero-inflated), high-dimensional, and extremely non-normal data.

### Pathway enrichment analysis

Enrichment analyses were performed using the *omePath* package^6^. *omePath* assigns an importance score (i.e., coefficient score from the CPLM model) to each omics feature (e.g., proteins, metabolites) and performs statistical tests (Wilcoxon sum rank) between rank of feature score in a given pathway against all ranks to calculate a p-value for the null hypothesis. There is no difference between the distribution of score of features with the pathways of interest vs. all other features in the study. We used an alpha level of 0.05 for significance. *omePath* is an open-source software implemented as an R package with code, tutorials, and documentation at https://github.com/omicsEye/omePath. The result for each association contains the identified pathway, members of the pathways, number of observed members in the study (n), and the total number of pathway members in the database (HMDB database for metabolite pathways^120^ and Reactome pathways database^121^ for proteins), used for the analysis, p-value, q-value from Benjamini–Hochberg FDR correction (q = 0.25).

### Machine learning algorithms

Machine learning (ML) algorithms like neural networks are often considered to be a black box because of their inability to provide a simple and straightforward explanation of their predictions. Nevertheless, this prediction model generally exceeds simple linear models or decision trees and random forest predictions. Yet, such simple models are still preferred in the field of medical science due to their simplicity and interpretability^169–171^. Many studies have been targeted to build and execute model-agnostic interpretability tools^172–174^. We use the term feature importance to explain how important a feature is to the model’s predictive performance. The most well-known approach is using permutation importance which was introduced by Breiman^175^. Using permutation importance, we have quantified the importance score of features for predicting health outcomes. The features consist of metabolomics, proteomics, and clinical info for all the patients considered in the study. The samples were labeled into four labels based on the health status; Healthy, non-COVID-19, COVID-19, and severe-COVID-19. We predicted the health status of a patient based on the various proteomics and metabolomics data and found the importance of each feature inside the prediction model such as beta-alanine and 15-HETE metabolites which both were ranked as top influential features in RF and DNN models (SFig. 5).

Data from a real-world scenario are never flawless. The medical records and the clinical information for the patients affected by COVID-19 are no exception. The data in this study contained many values which were lost due to machine or human error. The data thus was cleaned of all the unknown values and also the null values by dropping the instance with the missing feature value. Serum panel data tends to have a significant difference between the maximum and minimum value, to rectify this issue, we used normalization. Normalization is a scaling-down transformation in data where the difference between min and max values is significantly big. The data were normalized using a min–max scaler function which is present in the *sklearn* package.

### Decision tree

Decision Tree follows a flow-chart-like structure where the nodes are the features, the branches are the decision rules, and the leaves are the outcomes. Decision Tree is a supervised learning method that utilizes a divide and conquer approach; it selects the best attribute using Information Gain and then divides the dataset into a subset. This division is performed repeatedly until the method reaches a child node which satisfies the condition of no remaining attributes or no more remaining instances.

### KNN- K nearest neighbor

K-Nearest Neighbor (KNN) is a supervised machine learning technique that is dependent on the training dataset. The K, in KNN, stands for a user-defined number. This algorithm assumes that data points with similar features reside in close proximity to each other. Proximity is generally calculated in the form of euclidean distances among points. In a classification problem such as ours, the distances between the test data points and the training data points are calculated, sorted, and stored in a table^176^. Then, the mode of the labels of K- nearest neighbors using the sorted table is given as an output.

### Random forest

Random Forest is a supervised algorithm that randomly selects a subset of the training dataset and creates a decision tree on the subset; it then carries out a vote to predict the class of the test data points.

### Logistic regression

A predominant part of published propensity results uses Logistic Regression (LR). Logistic regression is a very sought after technique because of its mathematical ability to produce probability in the range [0,1]^177^. Logistic regression uses a functional approach to estimate the probability of binary response based on input features. LR finds the best-fit parameters to a nonlinear function called sigmoid^178^. Logistic regression models probability for a binary class, however, our health outcome variable has more than two classes. To address the binary class limitation of logistic regression, we used a ‘newton-cg’ solver. In our study, we use logistic regression as a baseline for the other methods.

### Deep neural network

Deep neural network (DNN) is a type of machine learning architecture that mimics the working of the neurons located in the brain, and how they transfer information to learn new problems for the purpose of solving them^179^. The inputs of DNN are fed in the input layers, which are passed through one or more hidden layers, which consist of neurons, where they are analyzed and processed to determine the output of the next layer. DNN uses a learning rule which correctly decides the weight and the bias of each neuron in the hidden layer and output layer. The power of DNN to determine and adapt the weight and bias dynamically makes it a powerful tool to capture the various complex and non-linear relationships among the various features, which in turn facilitates classification and prediction of correct labels, thus increasing the accuracy and efficiency of the model^180,181^.

We have incorporated the full extent of the data since some were discarded due to missing values. Including a more extensive set of data and features in deep learning, the model brings out a more comprehensive hidden complex relationships among all the proteins and metabolites. This enables a more accurate prediction and prioritization of metabolites and proteins for further studies to show how they affect a patient's health status. The importance scores for metabolites and proteins generated by the model are based on their degree of influence on the result.

The only ML model which was used in the above said paper was Random Forest (RF). Still, in our evaluation, we used decision tree (DT), k- nearest neighbors (KNN), random forest (RF), logistic regression (LR), and deep neural network (DNN). We have made a thorough comparison of all the methods using various metrics like accuracy, precision, recall, and F-1 score to evaluate the performance of each model. The accuracy of DNN comes higher than all other methods that were considered. We then used the DNN model for importance evaluation that leads to the discovery of numerous metabolites and proteins which when done, a thorough study shows a relationship with covid and health status.

The different evaluation metrics used in machine learning section are as follows:

$$\begin{aligned}&Accuracy = \frac{TN+TP}{FN+FP+TN+TP}\\&Precision = \frac{TP}{FP+TP}\\&Recall = \frac{TP}{FN+TP}\\&F-1 Score = \frac{2 * Precison *Recall}{Precision + Recall}\end{aligned}$$

## Supplementary Information

Supplementary Information 1.

Supplementary Information 2.

Supplementary Information 3.

Supplementary Information 4.
