# Supplementary material for: Metabolite, protein, and tissue dysfunction associated with COVID-19 disease severity
Source: Sci Rep. 2022 Jul 16;12:12204. doi: 10.1038/s41598-022-16396-9 (PMC9288092; doi:10.1038/s41598-022-16396-9)
Supplement: Supplementary file 1 — Supplementary Information 1. [file 41598_2022_16396_MOESM1_ESM.pdf]

Supplementary materials for

Metabolite, protein, and tissue dysfunction associated with COVID-19 disease severity

Ali Rahnavard[1]*, Brendan Mann[1,2], Abhigya Giri[1], Ranojoy Chatterjee[1], Keith A. Crandall[1]

[1]Computational Biology Institute, Department of Biostatistics and Bioinformatics, Milken Institute School of Public Health, The George Washington University, Washington, DC 20052

[2]Department of Microbiology, Immunology, and Tropical Medicine, School of Medicine and Health Sciences, The George Washington University, Washington, DC 20052

*Correspondence to rahnavard@gwu.edu

Supplementary Figures

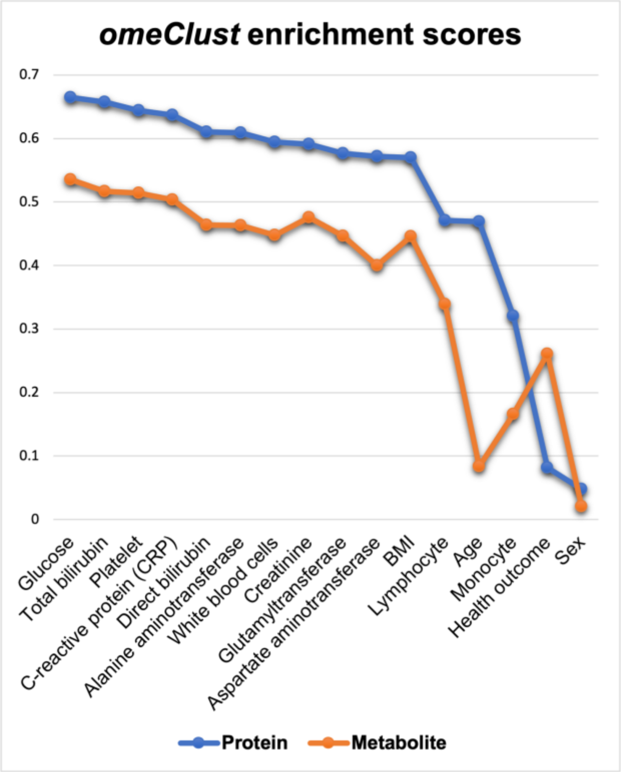

Supplementary Figure 1. omeClust[1] **enrichment score using unsupervised community detection reveals important clinical and patient information.** Bray-Curtis distance between samples was calculated for metabolite profiles and protein profiles. omeClust detected 7 major clusters (11 clusters were detached in total) using protein profiles and 6 major clusters (7 clustered were detached in total) using metabolites profiles. omeClust also measured an enrichment score for each metadata provides (16 clinical information and patients' characteristics). Glucose has the highest enrichment score, and Sex has the lowest. OmeClust uses normalized mutual information between discretized metadata and cluster labels to calculate enrichment scores.

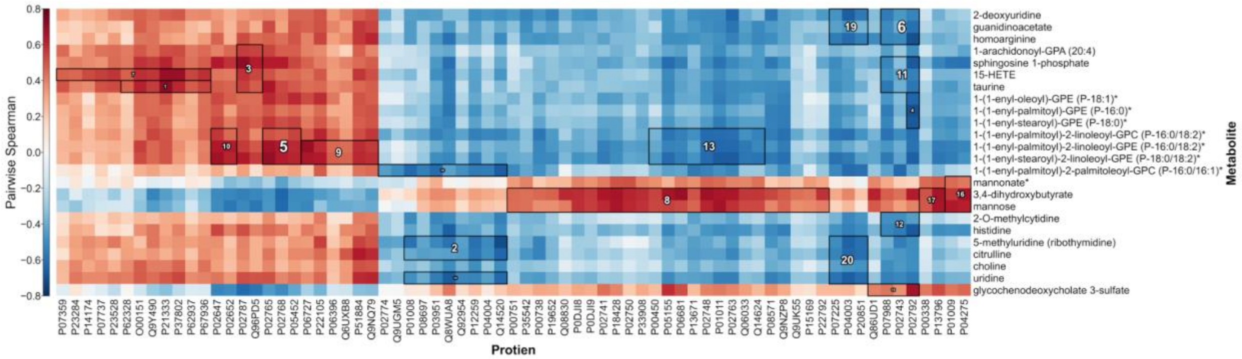

Supplementary Figure 2. Block-wise associations among metabolites and proteins. Correlation between metabolite and protein intensities across 83 paired samples was tested using *btest*. Block of features with FDR threshold 0.05 were reported significant. *btest* finds groups of features within each dataset using Spearman correlations and if all members of two groups are significantly associated then reports as a block of associations. Groups within each dataset are detected using clustering. Block associations are ranked based on the minimum p-value of each block. A block with the smallest p-value gets rank 1.

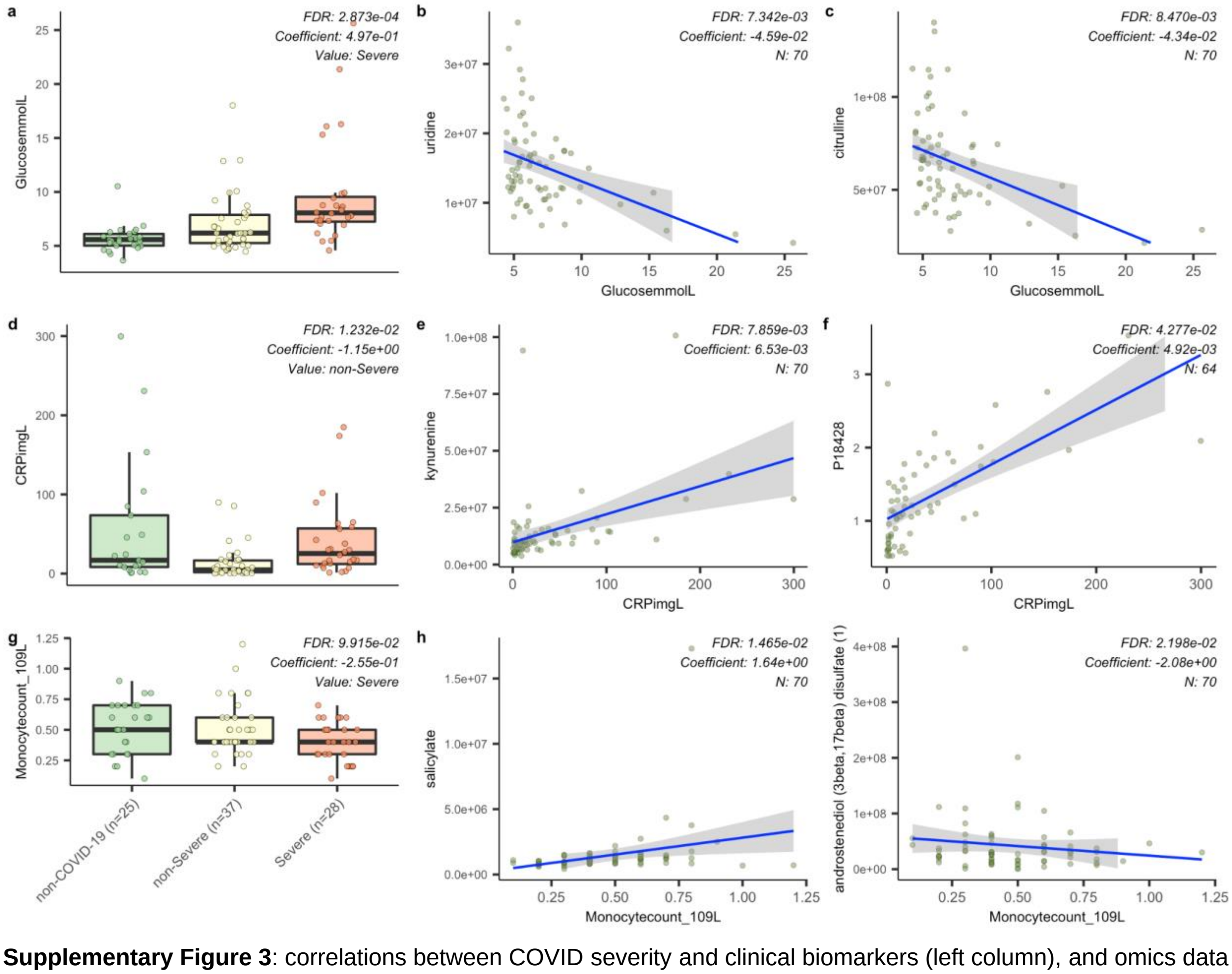

Supplementary Figure 3: correlations between COVID severity and clinical biomarkers (left column), and omics data and clinical outcome (middle and right columns) within the context of COVID-19. **a, d,** and **g** show clinical biomarker patterns at different COVID severity levels. **b, c, e, f, h,** and **i** show significant linear associations between clinical features and either a metabolite or a protein.

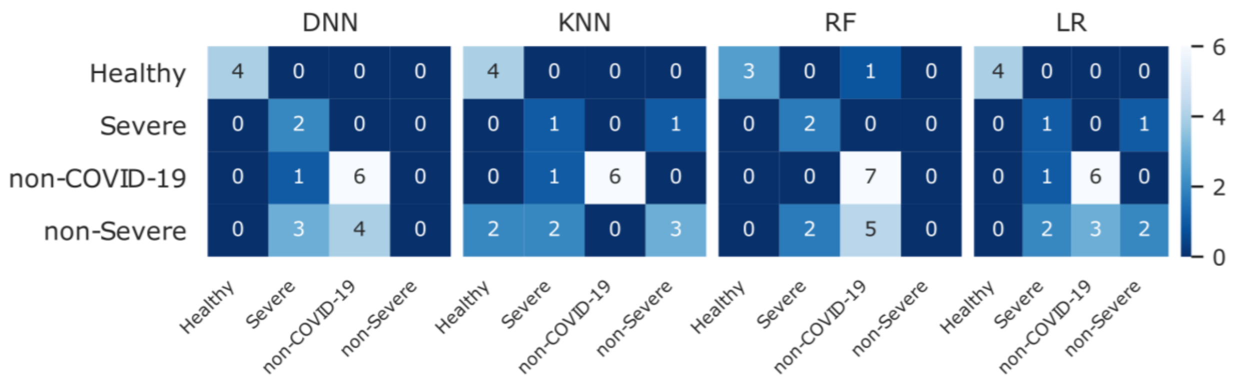

Supplementary Figure 4: The Confusion Matrix to show the accuracy of different methods. The confusion matrix of the ANN shows that it correctly identifies all the severe cases of COVID-19, where most of the other methods have some degree of error. It also has a lower error in detecting the Covid cases since it identifies them as Severe Covid and not healthy or Non-Covid.

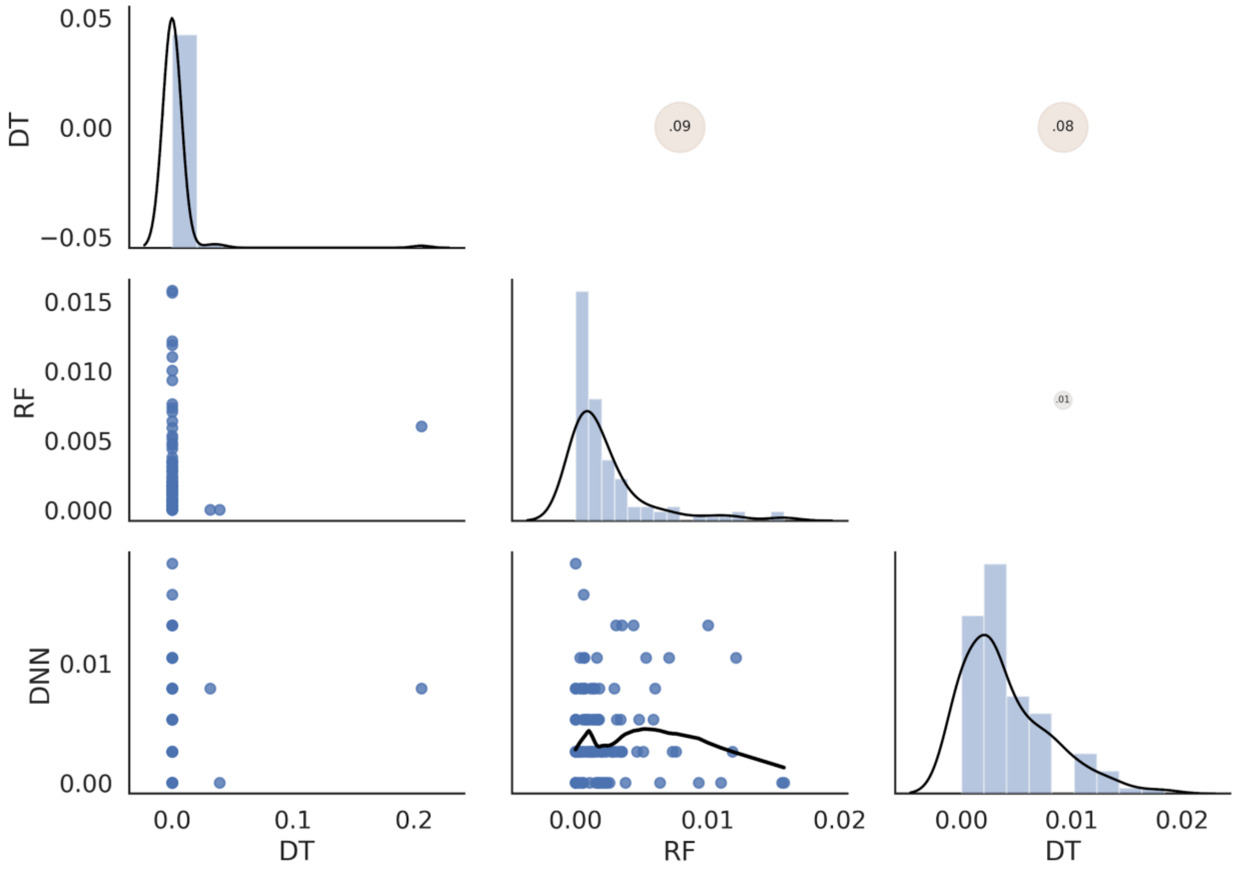

Supplementary Figure 5: Importance score from various machine learning techniques: there are several places where RF and DNN have produced equivalent results, such as beta-alanine and 15-HETE that provide high and correlated scores for metabolites.

Supplementary Tables

Biomarker	Pathway
Cytosine	Viral synthesis
Uridine	Liver impairment
Purine metabolism	Acute lung injury
*Citrulline	Multi-organ dysfunction
BCAAs	Protein anabolism
Benzoate	Proinflammatory and anti-inflammatory
Hyaluronan-binding protein 2 (FSAP, protein ID Q14520)	inflammation pathways in non-immune cell populations
Prenylcysteine oxidase 1 (PCYOX1 protein, protein ID Q9UHG3)	Liver function
15-HETE	inflammation
complement component 2 (C2, protein ID P06681)	B lymphocyte regulation, inflammation, and host protection
complement component 9 (C9, Protein ID P02748)	B lymphocyte regulation, inflammation, and host protection
histidine-rich glycoprotein (HRG, protein ID P04196)	immune system regulation, cell adhesion, angiogenesis, and coagulation
Lumican (protein ID P51884)	Fibril assembly and stromal collagen matrix assembly
Glucose	perhaps inflammation
**C-Reactive Protein (CRP)	Inflammation and response to infection
**Monocyte	Immune dysregulation
Salicylate	Non-steroidal anti-inflammatory drugs
Sphingomyelin	Cell-cell interactions and intracellular signaling

* biomarkers have been discussed in 1-5 publications as listed in the OncoMX COVID-19 Biomarker Database [2]<br>** biomarkers have been discussed in more than 20 publications as listed in the OncoMX COVID-19 Biomarker Database [2]

Supplementary Table 1: A summary table of discussed biomarkers and related health pathways.

Supplementary Table 2: Association testing results for metabolites and proteins vs. patients' characteristics and clinical information.

Supplementary Table 3: Pathway enrichment analysis results using metabolites and proteins for Severe, non-Severe, and non-COVID-19 group vs. healthy group.

Supplementary references

1. Rahnavard A, Chatterjee S, Sayoldin B, et al. Omics community detection using multi-resolution clustering. Bioinformatics 2021;
2. Gogate N, Lyman D, Bell A, et al. COVID-19 biomarkers and their overlap with comorbidities in a disease biomarker data model. Brief. Bioinform. 2021;
