# Supplementary material for: Metabolite, protein, and tissue dysfunction associated with COVID-19 disease severity
Source: Sci Rep. 2022 Jul 16;12:12204. doi: 10.1038/s41598-022-16396-9 (PMC9288092; doi:10.1038/s41598-022-16396-9)
Supplement: Supplementary file 2 — Supplementary Information 2. [file 41598_2022_16396_MOESM2_ESM.docx]

| **Biomarker** | **Pathway** |
| --- | --- |
| Cytosine | Viral synthesis |
| Uridine | Liver impairment |
| Purine metabolism | Acute lung injury |
| *Citrulline | Multi-organ dysfunction |
| BCAAs | Protein anabolism |
| Benzoate | Proinflammatory and anti-inflammatory |
| Hyaluronan-binding protein 2 (FSAP, protein ID Q14520) | inflammation pathways in non-immune cell populations |
| Prenylcysteine oxidase 1 (PCYOX1 protein, protein ID Q9UHG3) | Liver function |
| 15-HETE | inflammation |
| complement component 2 (C2, protein ID P06681) | B lymphocyte regulation, inflammation, and host protection |
| complement component 9 (C9, Protein ID P02748) | B lymphocyte regulation, inflammation, and host protection |
| histidine-rich glycoprotein (HRG, protein ID P04196) | immune system regulation, cell adhesion, angiogenesis, and coagulation |
| Lumican (protein ID P51884) | Fibril assembly and stromal collagen matrix assembly |
| Glucose | perhaps inflammation |
| **C-Reactive Protein (CRP) | Inflammation and response to infection |
| **Monocyte | Immune dysregulation |
| Salicylate | Non-steroidal anti-inflammatory drugs |
| Sphingomyelin | Cell-cell interactions and intracellular signaling |
| * biomarkers have been discussed in 1-5 publications as listed in the OncoMX COVID-19 Biomarker Database[^2^](https://paperpile.com/c/1VNPst/BOXz)  ** biomarkers have been discussed in more than 20 publications as listed in the OncoMX COVID-19 Biomarker Database[^2^](https://paperpile.com/c/1VNPst/BOXz) | |

**Supplementary** **Table 2:** A summary table of discussed biomarkers and related health pathways.
